# MALT1-Deficient Mice Develop Atopic-Like Dermatitis Upon Aging

**DOI:** 10.3389/fimmu.2019.02330

**Published:** 2019-10-01

**Authors:** Annelies Demeyer, Elien Van Nuffel, Griet Baudelet, Yasmine Driege, Marja Kreike, David Muyllaert, Jens Staal, Rudi Beyaert

**Affiliations:** ^1^VIB Center for Inflammation Research, Ghent, Belgium; ^2^Department of Biomedical Molecular Biology, Ghent University, Ghent, Belgium

**Keywords:** atopic dermatitis, skin inflammation, MALT1, lymphocytes, Tregs, Th2, aging

## Abstract

MALT1 plays an important role in innate and adaptive immune signaling by acting as a scaffold protein that mediates NF-κB signaling. In addition, MALT1 is a cysteine protease that further fine tunes proinflammatory signaling by cleaving specific substrates. Deregulated MALT1 activity has been associated with immunodeficiency, autoimmunity, and cancer in mice and humans. Genetically engineered mice expressing catalytically inactive MALT1, still exerting its scaffold function, were previously shown to spontaneously develop autoimmunity due to a decrease in Tregs associated with increased effector T cell activation. In contrast, complete absence of MALT1 does not lead to autoimmunity, which has been explained by the impaired effector T cell activation due to the absence of MALT1-mediated signaling. However, here we report that MALT1-deficient mice develop atopic-like dermatitis upon aging, which is preceded by Th2 skewing, an increase in serum IgE, and a decrease in Treg frequency and surface expression of the Treg functionality marker CTLA-4.

## Introduction

MALT1 (Mucosa-associated lymphoid tissue lymphoma translocation protein 1) is an intracellular signaling protein that plays an important role in several cell types, including lymphoid and myeloid cells as well as non-hematopoietic cells (1). MALT1 is best known for its role as a scaffold protein in T cell receptor (TCR)- and B cell receptor (BCR)-induced nuclear factor-κB (NF-κB) signaling, leading to the activation and proliferation of T and B cells, respectively (2, 3). Moreover, MALT1-mediated NF-κB signaling plays a key role in the proliferation of certain B cell lymphomas, such as MALT1 lymphoma and activated B cell-like diffuse large B cell lymphoma (ABC-DLBCL) (4–13). TCR or BCR stimulation, as well as oncogenic mutations in specific signaling proteins, leads to the formation of a so-called CBM signaling complex, consisting of CARD11 (also known as CARMA1), BCL10 and MALT1 (8, 14–18). In this complex, MALT1 acts as an adaptor to recruit the E3 ubiquitin ligase TRAF6, whose activity facilitates the recruitment and activation of downstream NF-κB signaling proteins (19–21). The importance of the CBM complex is illustrated by the impaired TCR-induced NF-κB activation in T cells isolated from *Card11*-*, Bcl10*-, and *Malt1*-knock-out (KO) mice, respectively (2, 3, 22, 23).

In addition to its scaffold function, MALT1 also acts as a cysteine protease. TCR stimulation leads to the MALT1-mediated cleavage of several substrates including BCL10, the deubiquitinases A20 and CYLD, the NF-κB family member RelB, the RNA-binding and RNA-destabilizing proteins Roquin-1/2, Regnase-1, and N4BP1, the E3 ubiquitin ligase HOIL1, and MALT1 itself (24–34). Although the specific biological role of cleavage of each of these substrates is still largely unclear, MALT1 proteolytic activity contributes to the fine-tuning of TCR-induced gene expression, lymphocyte activation and proliferation, and regulatory T cell (Treg) development and function. Consequently, inhibition of MALT1 proteolytic activity has been proposed as an interesting therapeutic approach for autoimmune diseases and certain cancers, which is further supported by promising results with MALT1 protease inhibitors in preclinical mouse models (12, 13, 35–37). Of note, MALT1 mutation in humans, causing the absence or very low expression of MALT1, leads to combined immunodeficiency (CID), which is characterized by several bacterial, fungal, and viral infections, indicating that targeting MALT1 activity may not be without risk (38–43). Moreover, it was recently shown that *Malt1* protease-dead (PD) knock-in mice expressing a catalytically inactive MALT1 mutant spontaneously develop multi-organ inflammation due to defects in T cell homeostasis (44–49). This was rather unexpected since inflammation was never described for mice that are completely deficient in MALT1. However, in the present paper we show that *Malt1*-KO mice develop atopic-like dermatitis upon aging.

## Results

### *Malt1*-KO Mice Spontaneously Develop Skin Lesions, Accompanied by Elevated Serum Cytokine Levels

**Figure 1:**
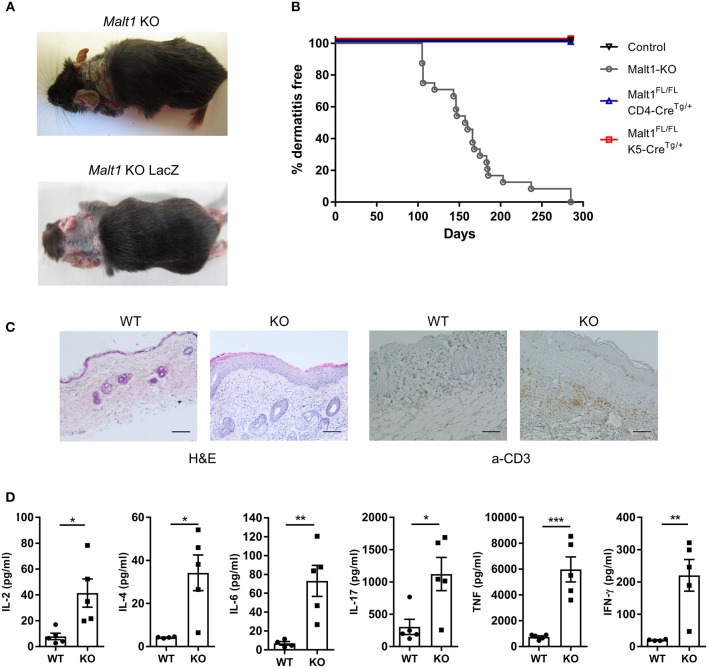
*Malt1*-KO mice develop skin lesions at late age accompanied by elevated serum cytokine levels. **(A)** Skin lesion in the neck of a *Malt1*-KO and *Malt1*-KO LacZ mouse. **(B)** Incidence of dermatitis in *Malt1*-KO mice (*n* = 8 for controls, *n* = 24 for *Malt1-KO, n* = 6 for *Malt1*^*FL*/*FL*^ *CD4-Cre*^*Tg*/+^, and *n* = 6 for *Malt1*^*FL*/*FL*^ *K5-Cre*^*Tg*/+^). **(C)** H&E staining on WT and *Malt1*-KO skin (scale bar 100 μm), showing epidermal thickening and parakeratosis in *Malt1*-KO skin and α-CD3 staining on WT and *Malt1*-KO skin (scale bar 100 μm), showing increased CD3 staining in *Malt1*-KO skin. **(D)** Elevated serum levels of IL-2, IL-4, IL-6, IL-17, IFN-γ, and TNF in *Malt1*-KO mice compared to WT mice (both *n* = 5 and >30 weeks, and *Malt1*-KO mice have AD). Open circles (WT) and black squares (KO) represent individual mice. The mean ^±^ SEM is indicated on the graphs. The statistical significance between groups was calculated with an unpaired 2-tailed Student's *t*-test: *P < 0.05, **P < 0.01, and ***P < 0.001.

**Figure 2:**
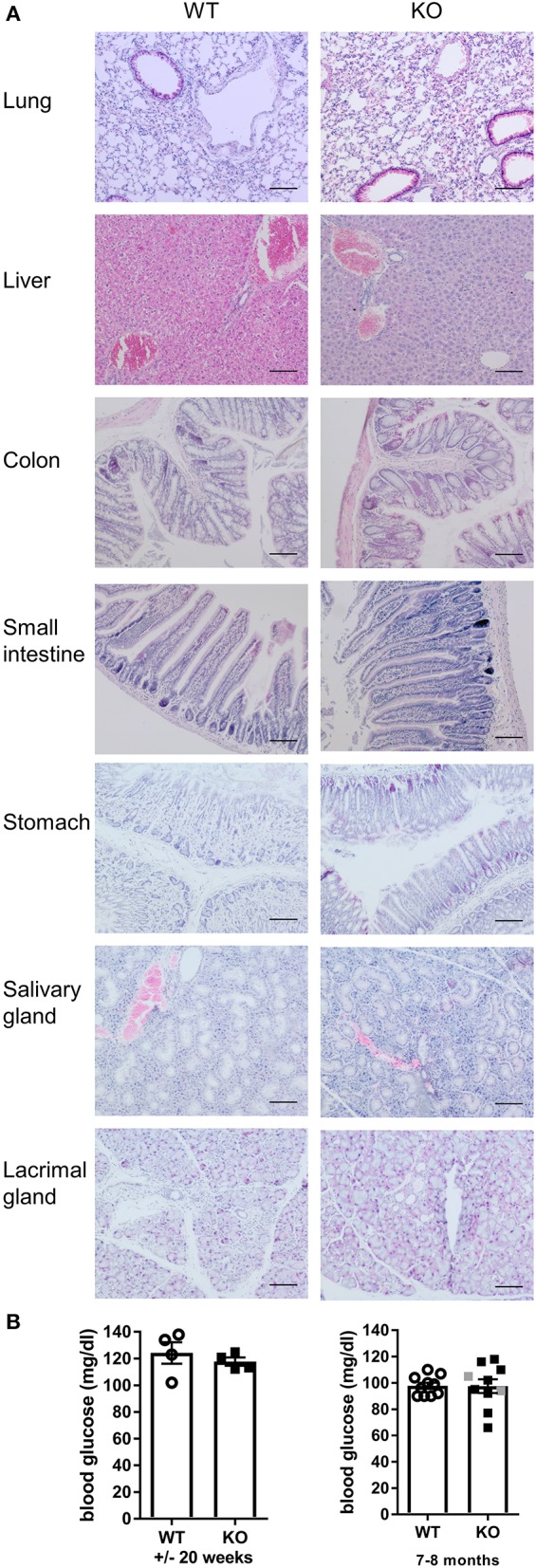
*Malt1*-KO mice do not develop multi-organ inflammation. **(A)** H&E staining (scale bar 100 μm) of several organs from WT and *Malt1*-KO mice (*Malt1-*KO mice had skin lesions and the WT and KO mice were between 30 and 40 weeks old). **(B)** Blood glucose levels in mice of ±20 weeks (left, WT: *n* = 5 and KO: *n* = 4) and mice of 7–8 months (right, WT: *n* = 10 and KO: *n* = 8). Open circles (WT), gray squares (KO), and black squares (KO + AD = KO mice with atopic dermatitis) represent individual mice. The mean ± SEM is indicated on the graphs. The statistical significance between groups was calculated with an unpaired 2-tailed Student's *t*-test: no significant difference was found.

*Malt1*-KO mice were housed under SPF conditions and monitored for macroscopic clinical signs on a regular basis. Interestingly, the mice were found to develop skin lesions upon aging, with an average disease onset of 161 days (Figures 1A,B). The *Malt1*-KO mice suffer from erosive lesions in the neck and face region, with the epidermis showing acanthosis, hyperkeratosis, and parakeratotic scaling, as well as CD3^+^ T cell infiltration (Figure 1C). Similar lesions were observed in another independent *Malt1*-KO LacZ reporter mouse line (Figure 1A), indicating that the observed phenotype is strain-independent. Next to full body *Malt1*-KO mice, also T cell-specific (*Malt1*^*FL*/*FL*^*CD4-Cre*^*Tg*/+^) and keratinocyte-specific (*Malt1*^*FL*/*FL*^*K5-Cre*^*Tg*/+^) *Malt1-KO* mice were monitored for skin lesions over time, but these mice did not develop any skin lesions (Figure 1B), indicating that absence of MALT1 in T cells or keratinocytes, as such, is not sufficient to induce skin inflammation. *Malt1-*KO mice that develop skin inflammation were found to have increased serum levels of the pro-inflammatory cytokines IL-2, IL-4, IL-6, IL-17, IFN-γ, and TNF (Figure 1D). To assess if increased serum cytokine levels reflect a more general inflammation in MALT1-deficient mice, we analyzed H&E stained sections of lung, liver, stomach, colon, small intestine, lacrimal glands and salivary glands. However, no differences were observed between *Malt1*-KO and WT mice for all these tissues (Figure 2A). In addition, we checked blood glucose levels in young (±20 weeks) and older mice (7–8 months) to determine possible pancreatic inflammation, but also here MALT1 deficiency had no effect (Figure 2B). Together, our data demonstrate that MALT1 deficiency in mice specifically results in an inflammatory skin phenotype upon aging.

### MALT1 Deficiency Results in Defective Treg Development and CTLA-4 Expression via a T Cell Intrinsic Mechanism in Both Young and old Mice

**Figure 3:**
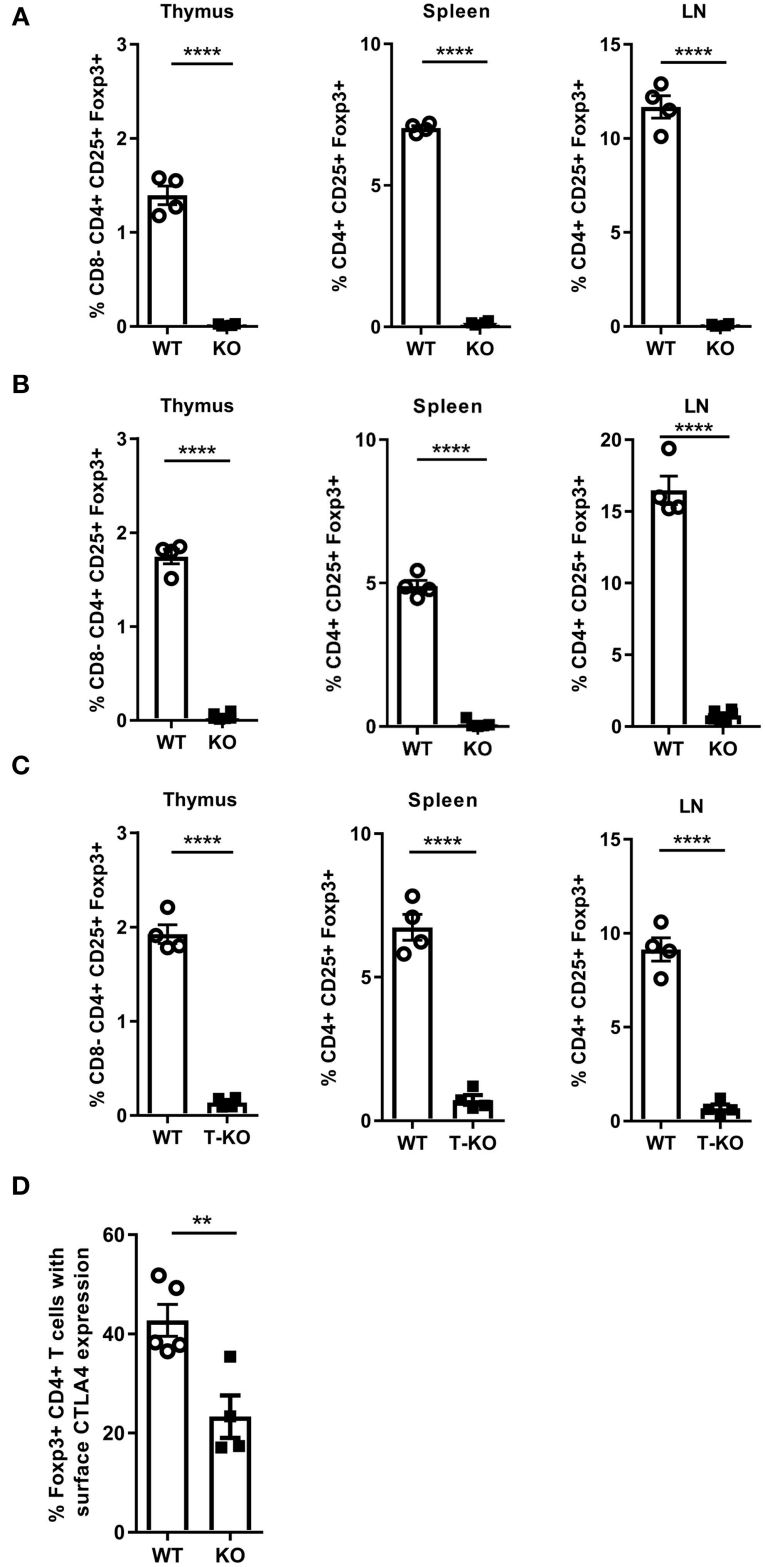
MALT1 plays a T cell-intrinsic role in Treg development. **(A)** Percentage of Tregs (Foxp3^+^CD25^+^CD4^+^ T cells) in the thymus, spleen and lymph nodes of *Malt1*-WT and *Malt1*-KO mice without skin lesions (WT: *n* = 4 and KO: *n* = 4, age 10–12 weeks). **(B)** Percentage of Tregs (Foxp3^+^CD25^+^ CD4^+^ T cells) in the thymus, spleen and lymph nodes of older WT and *Malt1-*KO mice with skin lesions (WT: *n* = 4 and KO: *n* = 6, age 6.5–8.5 months). **(C)** Percentage of Tregs (Foxp3^+^CD25^+^CD4^+^ T cells) in the thymus, spleen and lymph nodes of WT (*Malt1*^*FL*/*FL*^*CD4-Cre*^+/+^, *n* = 4), and T-KO *(Malt1*^*FL*/*FL*^*CD4-Cre*^*Tg*/+^, *n* = 4) mice. **(D)** Percentage of splenic Tregs (Foxp3^+^CD4^+^ T cells) expressing CTLA-4 on their surface after stimulation for 4 h with PMA and IO in WT (*n* = 5) and *Malt1-*KO (*n* = 5) mice. The mean ± SEM is indicated on the graphs. The statistical significance between groups was calculated with an unpaired 2-tailed Student's *t*-test: **P < 0.01 and ****P < 0.0001.

*Malt1-*KO mice are known to have a defect in Treg development (44, 46, 50, 51), which could be responsible for the skin inflammation in aging MALT1-deficient mice. However, it has been reported by Brüstle et al. that whereas young *Malt1*-KO mice have severely reduced numbers of Tregs in blood and thymus, 1 year old *Malt1*-KO mice have normal Treg numbers in blood, which was suggested to reflect the generation of inducible Tregs (iTregs) in aging mice (51). Of note, this study did not mention the development of skin lesions in aged mice. We therefore analyzed the number of Tregs (Foxp3^+^CD25^+^CD4^+^ T cells) in young and aged (±7 months old) *Malt1*-KO mice. In agreement with the above mentioned previous studies, Treg numbers were reduced in thymus, lymph nodes (LN), and spleen of lesion-free young mice. However, in contrast to the study by Brüstle et al., we found that the number of Tregs were equally reduced in thymus, LN and spleen of aged *Malt1*-KO mice that developed skin lesions (Figures 3A,B and gating strategy in Supplementary Figures 1, 2). The reason for this discrepancy is still unclear, but different findings may reflect specific differences in mouse housing conditions. Similar to the full *Malt1*-KO mice, also T cell specific *Malt1*-KO mice had a reduced Treg frequency in their thymus, spleen, and LN (Figure 3C), indicating a T cell intrinsic role of MALT1 in Treg development. We next investigated whether the remaining MALT1-deficient Tregs are functional. CTLA-4 expression on Tregs is known to compete with CD28 on T cells for binding to CD80 and CD86, as well as to reduce the surface expression of CD80 and CD86 on antigen presenting cells, resulting in reduced T cell proliferation and cytokine production (52, 53). We therefore assessed CTLA-4 surface expression on splenocytes from WT and *Malt1*-KO mice that were stimulated *in vitro* for 4 h with phorbol myristic acid/Ionomycine (PMA/IO). As shown in Figure 3D, a reduced frequency of Tregs that express surface CTLA-4 could be observed in *Malt1*-KO mice compared to WT mice, suggesting that the remaining MALT1-deficient Tregs are functionally impaired (gating strategy in Supplementary Figure 2).

### Activation and CTLA-4 Surface Expression of CD4^+^ T Cells Is Altered in *Malt1*-KO Mice

**Figure 4:**
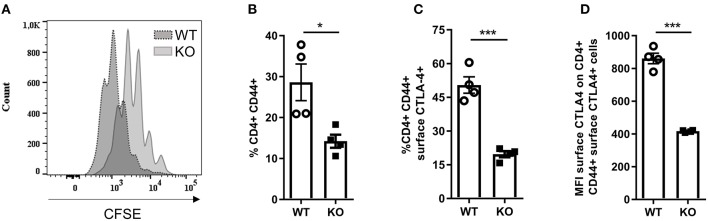
Activation of *Malt1*-KO CD4^+^ T is altered. **(A)** CFSE labeled CD4^+^ T cells stimulated for 72 h with plate bound α-CD3 and soluble α-CD28. The CFSE overlaid histograms displaying the proliferation of WT or *Malt1-*KO CD4^+^ T cells (age mice: 10 weeks lesion-free). **(B)** Splenocytes were stimulated for 4 h with PMA and IO (same for C and D) in order to determine the percentage of CD44^+^ CD4^+^ T cells. **(C)** Percentage of CD44^+^CD4^+^ T cells that express CTLA-4 on their surface. **(D)** Mean fluorescence intensity (MFI) of CTLA-4 on CD44^+^CD4^+^ T cells that express CTLA-4 on their surface. For **(B–D)** open circles (WT: *n* = 4) and black squares (KO: *n* = 4) represent individual mice. The mean ± SEM is indicated on the graphs. The statistical significance between groups was calculated with an unpaired 2-tailed Student's *t*-test: *P < 0.05 and ***P < 0.001.

Since we observed increased CD3^+^ T cell infiltration in the diseased skin of *Malt1*-KO mice, we further investigated whether the proliferation and activation of CD4^+^ T cells is affected in MALT1-deficient mice. For this purpose, we purified CD4^+^ T cells and labeled them with CFSE to measure their proliferation after 72 h stimulation with anti-CD3 and anti-CD28. This showed that MALT1-deficient CD4^+^ T cells can proliferate, albeit to a lesser extent than WT CD4^+^ T cells (Figure 4A), which is similar to what has previously been described (45). To assess the activation of CD4^+^ MALT1-deficient T cells, we stimulated splenocytes for 4 h with PMA/IO and determined the frequency of CD44^+^CD4^+^ T cells, so-called effector CD4^+^ T cells. Notably, *Malt1*-KO mice had a reduced frequency of effector CD4^+^ T cells (Figure 4B), which is consistent with previous findings (2).

Since not only Tregs but also effector CD4^+^ T cells can use surface CTLA-4 to suppress proliferation of effector CD4^+^ T cells (54–56), we assessed the expression of CTLA-4 on the surface of effector CD4^+^ T cells from *Malt1*-KO and WT mice. In contrast to WT mice, *Malt1*-KO mice showed a strong reduction in the frequency of effector CD4^+^ T cells that express surface CTLA-4 (Figure 4C and gating strategy in Supplementary Figure 2). In addition, the expression of CTLA-4 on the remaining MALT1-deficient surface CTLA-4^+^ effector CD4^+^ T cells was also reduced, as determined by the surface CTLA-4 mean fluorescent intensity (Figure 4D). These data clearly show that besides being important for CTLA-4 expression on the surface of Tregs, MALT1 is similarly important for the expression of CTLA-4 on the surface of effector CD4^+^ T cells. Together, these data show that although MALT1 deficiency leads to reduced activation and proliferation of stimulated CD4^+^ T cells, it also lowers the immune suppressive functions of both Tregs and effector CD4^+^ T cells, which could contribute to disease development.

### MALT1 Deficiency Causes Th2 Skewing Accompanied by Increased Serum IgE Levels

**Figure 5:**
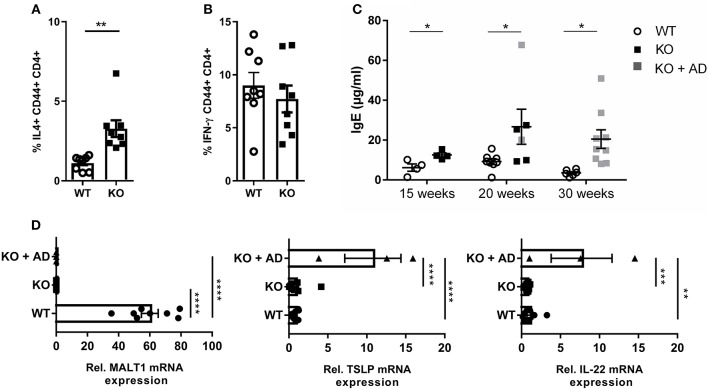
Th2 skewing and elevated IgE levels in *Malt1*-KO mice. **(A)** Splenocytes of 20 weeks old mice without skin lesions were stimulated for 4 h with PMA and IO (same for B), in order to determine the percentage of CD44^+^CD4^+^ T cells expressing IL-4 (WT and KO: *n* = 8). **(B)** Percentage of CD44^+^ CD4^+^ T cells expressing IFN-γ (WT and KO: *n* = 8). **(C)** IgE levels in serum collected from WT and *Malt1*-KO mice of ±15, ±20, and >30 weeks (15 weeks WT and KO: *n* = 4, at 20 weeks WT: *n* = 8 and KO: *n* = 6 and at 30 weeks WT: *n* = 6 and KO: *n* = 9). Open circles (WT), black squares (KO), and gray squares (KO + AD) represent individual mice. **(D)** mRNA expression levels of *Malt1, Tslp*, and *IL22* (relative to reference genes *Hprt1* and *Ubc*) in ears of *Malt1*-WT mice (*n* = 7), *Malt1*-KO mice (*n* = 11), and *Malt1*-KO + AD mice (*n* = 3) suffering from dermatitis. The mean ± SEM is indicated on the graphs. For **(A–C)** all results were obtained by flow cytometry the statistical significance between groups was calculated with an unpaired 2-tailed Student's *t*-test. For **(D)** the statistical significance between groups was calculated with a one-way ANOVA with a Tukey's multiple comparison test. *P < 0.05, **P < 0.01, ***P < 0.001, and ****P < 0.0001.

We next investigated whether the impaired Treg development in *Malt1*-KO mice has an impact on the T-helper (Th) cell populations. To this end, we stimulated splenocytes with PMA, IO, and Brefeldin A for 4 h, and determined the percentage of Th2 (IL-4 producing CD44^+^CD4^+^ T cells) (Figure 5A) and Th1 cells (IFN-γ producing CD44^+^CD4^+^ T cells) (Figure 5B), respectively (gating strategy in Supplementary Figure 3). MALT1-deficient mice (±20 weeks, skin lesion free) repeatedly showed largely similar levels of IFN-γ-producing Th1 cells, while the IL-4 producing Th2 cells were significantly increased. Since IL-4, secreted by Th2 cells, is known to induce B cell Ig isotype switching from IgM to IgE (57), we determined the serum IgE levels from *Malt1*-KO and WT mice of several ages. In agreement with the increased Th2 frequency, IgE levels were clearly elevated in *Malt1*-KO mice at any time point tested and preceded lesion onset (Figure 5C). Furthermore, in the ear skin of *Malt1-*KO mice with lesions, we found elevated mRNA levels of *Tslp* and *Il22* (Figure 5D), which are both known to promote Th2 responses (58, 59). Collectively, these data indicate that MALT1 deficiency leads to Th2 skewing and IgE production, which might contribute to skin lesion development.

## Discussion

We report that aging *Malt1*-KO mice suffer from atopic-like dermatitis accompanied by elevated serum cytokine levels and preceded by Th2 skewing and elevated serum IgE levels. No inflammation could be observed at other sites of the body. In contrast to *Malt1*-KO mice, skin lesions were never reported in mice fully deficient in one of the other components of the CBM complex, BCL10 and CARD11, or in the upstream activator PKCθ, even though BCL10 and PKCθ deficient mice were followed up until 6 months of age (22, 60). Notably, despite being part of the same signaling pathway, MALT1, BCL10, and PKC-θ deficiency have also been reported to differentially affect TCR-induced proliferation, IL-2 production, and NF-κB activation in T cells, with MALT1-deficient T cells showing a milder impairment than BCL10- and PKCθ-deficient T cells, suggesting they may have divergent functions and act in additional signaling pathways (61).

We further report that atopic-like dermatitis in aging *Malt1*-KO mice is associated with a decrease in the number and function of Tregs in the thymus and periphery. We propose that the reduction in immune suppressive Tregs leads to a disruption of normal immune homeostasis and contributes to the activation of effector T cells and allergic skin inflammation. In this context, we could measure more Th2 cells producing IL-4, which is known to play multiple roles in promoting atopic-like dermatitis (62). A severe Treg reduction is also seen in patients suffering from immunodysregulation polyendocrinopathy enteropathy X-linked (IPEX) syndrome (OMIM #304790), caused by mutations in the *FOXP3* gene (63) as well as mice with a mutation in the *Foxp3* gene, so called scurfy mice (64). The scurfy mice and the IPEX patients illustrate variable autoimmune disorders. IPEX patients can suffer from type 1 diabetes mellitus and thyroid disease, increased IgE levels, asthma and food allergies, while dermatitis and increased IgE levels are also present in the scurfy mice (63, 65–67). A lack of functional Tregs is a common feature in *Malt1*-KO mice, scurfy mice and IPEX patients (44, 46, 50, 51, 68–70). Moreover, a scurfy-like phenotype was described for mice (*Malt1*^*FL*/*FL*^*Foxp3-cre*^*Tg*/+^) with a specific deletion of *Malt1* in Tregs (36, 49). However, while *Malt1*-KO mice as well as MALT1 CID patients display impaired T cell activation (3, 22, 38–47), this is not the case for IPEX patients, scurfy mice and mice only lacking *Malt1* in Tregs, where there is a failure to control T cell activation due to the absence of Tregs or the reduced functionality of Tregs leading to lymphoproliferation and autoimmunity, resulting in death (36, 49, 63, 65–67, 71).

CTLA-4 is a known functionality marker on Tregs and is required for their inhibitory function (52, 53). We show that the remaining Tregs in *Malt1*-KO mice are also functionally impaired as demonstrated by a reduced CTLA-4 expression. The reduced Treg frequency and functionality we observed in *Malt1*-KO mice is associated with an increased Th2 frequency. Th2 cells were previously shown to expand disproportionally upon depletion of Tregs, which tightly control the Th2 population via induction of apoptosis of Th2, but not Th1 cells (72). Moreover, Tian et al. showed that addition of recombinant CTLA-4-Ig to Treg depleted mice induces Th2 apoptosis and thus reduces the Th2 expansion, illustrating the tight control by Tregs on the Th2 population (72). In addition to Tregs, also effector CD4^+^ T cells make use of surface CTLA-4 to inhibit effector CD4^+^ T cells, albeit with much lower efficiency than Tregs (54–56). Notably, we show that CTLA-4 expression is also reduced on effector CD4^+^ T cells in *Malt1*-KO mice, which may also contribute to disease pathogenesis. Interestingly, CTLA-4 mRNA is post-transcriptionally regulated by the endonuclease Regnase-1 and the RNA-binding proteins Roquin-1 and-2, which were shown to be inactivated by MALT1-mediated cleavage, leading to stabilization of CTLA-4 and many other mRNA molecules (28, 29). Most likely, reduced CTLA4 expression in MALT1-deficient Tregs and effector CD4^+^ T cells reflects the absence of Regnase-1 and Roquin cleavage, leading to CTLA-4 mRNA degradation and reduced CTLA-4 protein expression.

Mice that are completely deficient in MALT1 as well as mice expressing a catalytically inactive (protease-dead) mutant MALT1 have a reduced number of Tregs, but only *Malt1*-PD mice develop severe autoimmune symptoms (44–51). Impaired TCR-mediated effector T cell activation, normally mediated by the MALT1 scaffold function, has been proposed to prevent spontaneous inflammation in full *Malt1*-KO mice (1). Our present finding that *Malt1*-KO mice spontaneously develop skin inflammation upon aging, implicates a role for MALT1-independent antigen or cytokine receptor signaling leading to low or intermediate T cell activation. The reduced frequency of functional Tregs in combination with a lowered effector T cell activation causes a gradual and selective expansion of Th2 cells, culminating in allergic skin inflammation without autoimmunity or generalized inflammation.

Skin inflammation in atopic dermatitis is assumed to arise due to a misdirected immune response against harmless antigens on the one hand, and to skin barrier defects on the other hand (73). We propose that scratching may cause local skin barrier defects, which in combination with the Treg deficiency and the Th2 skewing favors the specific development of skin lesions in *Malt1*-KO mice. This is further supported by a report showing that tape stripping in combination with Treg depletion results in skin thickening, increased IL-4 and IL-13 mRNA levels in the skin, and elevated serum IgE levels (74). Of note, whereas an increase in IL-4 was already detectable in *Malt*1-KO mice before the development of skin lesions, elevated levels of *Tslp* and *Il22* mRNA, which are known to promote expression of Th2 cytokines, such as IL-4, could only be detected in lesional ear skin. A possible explanation might be that increased *Tslp* and *Il22* levels only occur upon skin barrier disruption in skin lesions, which is in agreement with an observed increase in TSLP expression upon tape stripping (75, 76).

Persistent severe dermatitis (7/9) and increased serum IgE levels (4/8) have been described in patients with loss of function mutations in MALT1 (38, 40–43). Similarly, dermatitis has been reported in genome-wide association studies for *CARD11* (77) and is also one of the clinical features found in most patients with loss of function mutations in *CARD11* (78–80). Also mice that have hypomorphic mutations in *Card11* display dermatitis, reduced Tregs, and increased IgE and Th2 levels (81–83). The proposed mechanism for disease development is the tight relationship between Tregs and Th2 cell levels (72, 82). However, our T cell-specific *Malt1-*KO mice did not develop skin lesions, suggesting that absence of MALT1 in T cells only is not sufficient to drive skin inflammation in aging mice. CARD11 is a member of the CARD-CC protein family, which also contains CARD9, CARD10 (also known as CARMA3), and CARD14 (also known as CARMA2) (84), which can all form distinct CBM complexes in a cell-type specific manner. Recently, Peled at al. reported that two loss-of-function mutations in *CARD14* are associated with atopic dermatitis (85). CARD14 is mainly expressed in keratinocytes and activates MALT1 signaling in keratinocytes (86, 87), which led us hypothesize that MALT1 deficiency in keratinocytes was driving atopic-like dermatitis. However, we also did not observe any skin lesions in keratinocyte-specific *Malt1*-KO mice. Possibly, combined deficiency in T cells and keratinocytes is needed to induce the atopic skin phenotype in aged mice. Alternatively, we cannot exclude a role for other cell types as well.

In general, the here established relationship between impaired MALT1-dependent TCR signaling, partial Treg deficiency, and dysregulated accumulation of Th2 cells, may provide a mechanistic basis to explain the allergic responses in patients carrying *MALT1* and *CARD11* mutations, and invites future studies investigating associations between atopy and genetic variations in other components of the TCR-MALT1 signaling pathway.

## Materials and Methods

### Mice

*Malt1*-KO mice (backcrossed for more than 10 generations into C57BL/6 background) were a kind gift from Dr. T. Mak (Toronto, Canada). Another *Malt1* allele from EUCOMM (*Malt1*^*tm*1*a*(*EUCOMM*)*Hmgu*/+^) was derived from ES cells and subsequently back-crossed to germline-expressing *Flpe*-deleter mice (88) to generate a conditional *Malt1*-deficient allele (*Malt1*^*FL*/+^). The ES cells were also backcrossed to a germline-expressing *Cre*-deleter mouse (89) to obtain an alternative full deficient allele with a LacZ reporter (*Malt1*^*IRES*−*LacZ*/+^). To generate a T-cell specific knock-out, *Malt1*^*FL*/+^ was further crossed to *CD4-Cre*^*Tg*/+^ mice (90) and *K5-Cre*^*Tg*/+^ (91) and offspring was inter-crossed to select for Flpe-deleter-negative T cell-specific (*Malt1*^FL/FL^*CD4-Cre*^*Tg*/+^) and skin-specific (*Malt1*^FL/FL^ *K5-Cre*^*Tg*/+^) MALT1-deficient mice. *CD4-Cre* is always kept heterozygote by selecting one parent *CD4-Cre*^*Tg*/+^ and the other parent as *Cre*-negative. The specificity of *CD4-Cre* was confirmed via western blot (Supplementary Figure 4) using rabbit monoclonal anti-MALT1 (SC-28246, Santa Cruz) and anti-Cre (6905-3, Merck Millipore). The *K5-Cre* is always kept heterozygote by selecting a male *K5-Cre*^*Tg*/+^ and a female as *Cre*-negative, to avoid female germline transmission (91). Mice were housed in individually ventilated cages in a specific pathogen-free (SPF) facility. Mice were supplied with water and food *ad libitum* and experiments were performed in compliance with the guidelines of the University of Ghent Ethics Committee for the use of laboratory animals (EC2011-024 and 2013-066). Mice were monitored regularly for signs of dermatitis, consisting of hair loss in the facial, ear and neck region, together with redness, skin thickening, and scratching.

### Genotyping

For *Malt1*-KO-mice we used the primers MALT1-F (GTGCTCTTGTAATTTTCTGTGCTC), MALT1 WT-R (GGGTACATCATGGCCTGAACAGTTG), and MALT1 KO-R (GGGTGGGATTAGATAAATGCCTGCTC), resulting in 172 bp (WT) and 272 bp (KO) PCR products. The genotypings were made using GoTaq Green Hot Start (Promega) master mix, with a typical PCR program: 5 min 94°C denaturation, 35–40 cycles [45 s 94°C|30 s 60°C|45 s 72°C] and 10 min 72°C final elongation.

For the *Malt1*^*tm*1*a*(*EUCOMM*)*Hmgu*/+^ derived mice we monitored the *Malt1* Flox-allele or KO allele with the primers MALTcKO-F (GTTTCTCAGGTCTTTAGTTCATGTC), CoMLT-3-R (TATACTCTACATCTCCATGGT), MALTcKO-R (TTGTTTTGCAGATCTCTGCC), and MLT-LacZ-F (TCGCTACCATTACCAGTTGGT) resulting in 280 bp (WT), 400 bp (FL), 345 (KO), or 514 bp (KO-LacZ) PCR products. *Flp* was detected with the primers Flp-F (TTAGTTCAGCAGCACATGATG) and Flp-R (GGAGGATTTGATATTCACCTG), resulting in a 370 bp PCR fragment. K5-Cre was detected with the primers Cre-F (TGCCACGACCAAGTGACAGCAATG) and Cre-R (AGAGACGGAAATCCATCGCTCG) producing a 374 bp PCR fragment. CD4-Cre was detected with primers CD4-Cre-R (TCAAGGCCAGACTAGGCTGCCTAT) and CD4-Cre-F2 (TCTCTGTGGCTGGCAGTTTCTCCA) producing a 300 bp PCR fragment. The genotypings were made using the GoTaq Green Hot Start (Promega) master mix, with a typical PCR program: 5 min 95°C denaturation, 35–40 cycles [30 s 95°C|30 s 55–60°C|60 s 72°C] and 10 min 72°C final elongation.

### Histology and Immunohistochemistry

Skin, lung, liver, colon, small intestine, stomach, lacrimal gland, and salivary gland samples were fixed with 4% paraformaldehyde and imbedded in paraffin. Sections (5 μm) were stained with hematoxylin and eosin. Skin sections were also stained with anti-CD3 (clone CD3-12; Serotec). Images were acquired with a BX51 discussion microscope (Olympus) with PixeLink camera under 100× magnification.

### RNA Extraction, cDNA Synthesis, and Quantitative Real-Time PCR

After sacrifice, ears were collected and incubated overnight in RNA later at 4°C before long term storage at −70°C. For RNA extraction, the ears were transferred to TRIzol reagent (Invitrogen) and homogenized using the Precellys 24 (Bertin technologies with CK26 beads). After phenol/chloroform phase separation, RNA was isolated using the Aurum total RNA mini kit (Bio-Rad). cDNA was synthesized using the SensiFAST™ cDNA synthesis kit (Bioline), according to manufacturer's instructions. Quantitative PCR was done with a LightCycler 480 (Roche) using sensiFAST™ SYBR No-ROX kit (Bioline) with a total of 10 ng cDNA and 300 nM of specific primers in a 10 μl reaction. Real-time reactions were done in triplicates. The following specific primers were used (5′-3′): *Hprt1* Fwd AGTGTTGGATACAGGCCAGAC and *Hprt* Rev CGTGATTCAAATCCCTGAAGT; *Ubc* Fwd AGGTCAAACAGGAAGACAGACGTA and *Ubc* Rev TCACACCCAAGAACAAGCACA; *Malt1* Fwd GGACAAAGTCGCCCTTTTGAT and Rev TCCACAGCGTTACACATCTCA; *Il22* Fwd AGACAGGTTCCAGCCCTACAT and *Il22* Rev TCTTCTGGATGTTCTGGTCGT; *Tslp* Fwd TCTCAGGAGCCTCTTCATCCT and *Tslp* Rev CTCACAGTCCTCGATTTGCT. Analysis was done with qBase software (Biogazelle). Values were normalized to two reference genes, as determined by Genorm analysis.

### Blood Glucose Levels

A drop of blood from the tail was applied to a test strip and the glucose level was measured with a Freestyle lite glucose meter (Abbot).

### Flow Cytometry

#### Detection of Tregs

Single cell suspensions of thymus, spleen, and lymph nodes were surfaced stained with Aqua Live/dead fixable stain (Life Technologies) or Fixable Viability Dye eFluor 506 (eBioscience), anti-CD16/CD32 Fc block (clone 2.4G2; BD Biosciences), anti-CD3-V450 (clone 17A2; BD Biosciences) or anti-CD3 eFluor450 (clone 17A2; eBioscience), anti-CD4-FITC (clone GK1.5; BD Biosciences or eBioscience), anti-CD25-PercPcy5.5 (clone PC61; BD Biosciences) for 20 min. Next, cells were permeabilized for 30 min, followed by 30 min of intracellular staining for anti-Foxp3-PE (clone FJK-16s; eBioscience). For the intracellular staining, the Foxp3 buffer set (eBioscience) was used and all incubation steps were done on ice.

#### CTLA-4 Expression of Tregs and CD44^+^CD4^+^ Effector T Cells

Splenocytes cultured in complete medium (RPMI 1640 medium supplemented with 10% FCS, Sodium Pyruvate, L-glutamine, antibiotics, and 2-Mercaptoethanol) were stimulated with PMA (50 ng/ml) and ionomycin (IO) (1 μg/ml) for 4 h at 37°C. The cells were stained as mentioned above, but anti-CD44-APC-efluor780 (clone IM7; eBioscience) and anti-CTLA-4 PE-eFluor610 (clone UC10-4B9; eBioscience) were also included in the surface staining.

#### Analysis of Cytokines by Intracellular Cytokine Staining

Splenocytes were cultured in complete medium and stimulated with PMA (50 ng/ml), IO (500 ng/ml) and Brefeldin A (1 μg/ml) for 4–5 h at 37°C. Stimulated cells were washed, surface stained with anti-CD16/CD32, Aqua Live/dead fixable stain or Fixable Viability Dye eFluor 506, anti-CD3-v450 or anti-CD3 eFluor450, anti-CD4-FITC, APC-anti-CD44-APC eFluor780, for 20 min. Next, cells were fixed and permeabilized for 30 min. using the Foxp3 buffer set, followed by intracellular staining with anti-IL4-APC (clone 11B11; eBioscience) and anti-IFNγ-PE-Cy7 (clone XMG1.2; BD Pharmingen) for 30 min.

#### Proliferation of CD4^+^ T Cells

CD4^+^ T cells isolated with the MACS CD4^+^ T cell isolation kit II were labeled with 2.5 μM CellTrace™ CFSE (Life Technologies) according to the manufacturer's protocol. The cells were cultured in complete medium for 72 h with 5 μg/ml plate bound anti-CD3 (145-2C11; BD Pharmingen) and 1 μg/ml soluble anti-CD28 (37.51; BD Pharmingen) and 50 IU/ml recombinant mIL-2 (PSF, VIB). Cells were surface stained with Aqua Live/dead fixable stain, anti-CD4-FITC and fixed as mentioned above.

All data were obtained with a LSRII flow cytometer (BD Biosciences) and FlowJo Software (Treestar, Inc, Ashland, Ore) was used for data analysis.

### Analysis of IgE and Cytokines in Serum

Peripheral blood samples were collected for serum preparation. The level of IgE in serum was determined using the mouse IgE ELISA Ready-SET-Go kit (eBioscience) and the concentration of IgE was calculated using GraphPad Prism 6 (GraphPad Software, Inc). The levels of IL-2 (171-G5003M), IL-4 (171-G5005M), IL-6 (171-G5007M), IL-17 (171-G5013M), IFN-γ (171-G5017M), and TNF (171-G5023M) was determined by Bio-Plex (Biorad) according to the manufacturer's conditions.

### Statistical Analysis

Statistical analysis (indicated in the figure legends) was performed with GraphPad Prism 7.

## Data Availability Statement

All datasets generated for this study are included in the manuscript/Supplementary material.

## Ethics Statement

The animal study was reviewed and approved by University of Ghent Ethics Committee for the use of laboratory animals (EC2011-024 and 2013-066).

## Author Contributions

AD, DM, JS, and RB designed the experiments. AD performed all the experiments, except for Figure 1C (done by DM). EV and GB provided the technical assistance for Figure 5D and YD provided the technical assistance for Figure 3B. YD and MK assisted with the genotyping of mice. AD, JS, and RB contributed to the scientific discussion and wrote the manuscript.

### Conflict of Interest

The authors declare that the research was conducted in the absence of any commercial or financial relationships that could be construed as a potential conflict of interest.

## References

[1] Demeyer A Staal J Beyaert R Targeting MALT1 proteolytic activity in immunity, inflammation and disease: good or bad? Trends Mol Med. (2016) 22:135–50. 10.1016/j.molmed.2015.12.004 26787500

[2] Ruland J Duncan GS Wakeham A Mak TW Differential requirement for Malt1 in T and B cell antigen receptor signaling. Immunity. (2003) 19:749–58. 10.1016/S1074-7613(03)00293-0 14614861

[3] Ruefli-Brasse AA French DM Dixit VM. Regulation of NF-kappaB-dependent lymphocyte activation and development by paracaspase. Science. (2003) 302:1581–4. 10.1126/science.1090769 14576442

[4] Akagi T Motegi M Tamura A Suzuki R Hosokawa Y Suzuki H. A novel gene, MALT1 at 18q21, is involved in t(11;18)(q21;q21) found in low-grade B-cell lymphoma of mucosa-associated lymphoid tissue. Oncogene. (1999) 18:5785–94. 10.1038/sj.onc.1203018 10523859

[5] Dierlamm J Baens M Wlodarska I Stefanova-Ouzounova M Hernandez JM Hossfeld DK. The apoptosis inhibitor gene API2 and a novel 18q gene, MLT, are recurrently rearranged in the t(11;18)(q21;q21) associated with mucosa-associated lymphoid tissue lymphomas. Blood. (1999) 93:3601–9. 10339464

[6] Morgan JA Yin Y Borowsky AD Kuo F Nourmand N Koontz JI. Breakpoints of the t(11;18)(q21;q21) in mucosa-associated lymphoid tissue (MALT) lymphoma lie within or near the previously undescribed gene MALT1 in chromosome 18. Cancer Res. (1999) 59:6205–13. 10626814

[7] Streubel B Lamprecht A Dierlamm J Cerroni L Stolte M Ott G. T(14;18)(q32;q21) involving IGH and MALT1 is a frequent chromosomal aberration in MALT lymphoma. Blood. (2003) 101:2335–9. 10.1182/blood-2002-09-2963 12406890

[8] Lucas PC Yonezumi M Inohara N McAllister-Lucas LM Abazeed ME Chen FF. Bcl10 and MALT1, independent targets of chromosomal translocation in malt lymphoma, cooperate in a novel NF-kappa B signaling pathway. J Biol Chem. (2001) 276:19012–9. 10.1074/jbc.M009984200 11262391

[9] Ferch U Kloo B Gewies A Pfander V Duwel M Peschel C. Inhibition of MALT1 protease activity is selectively toxic for activated B cell-like diffuse large B cell lymphoma cells. J Exp Med. (2009) 206:2313–20. 10.1084/jem.20091167102109c 19841089 PMC2768866

[10] Hailfinger S Lenz G Ngo V Posvitz-Fejfar A Rebeaud F Guzzardi M. Essential role of MALT1 protease activity in activated B cell-like diffuse large B-cell lymphoma. Proc Natl Acad Sci USA. (2009) 106:19946–51. 10.1073/pnas.0907511106 19897720 PMC2785272

[11] Hu S Du MQ Park SM Alcivar A Qu L Gupta S. cIAP2 is a ubiquitin protein ligase for BCL10 and is dysregulated in mucosa-associated lymphoid tissue lymphomas. J Clin Invest. (2006) 116:174–81. 10.1172/JCI25641 16395405 PMC1323253

[12] Fontan L Yang C Kabaleeswaran V Volpon L Osborne MJ Beltran E. MALT1 small molecule inhibitors specifically suppress ABC-DLBCL *in vitro* and *in vivo*. Cancer Cell. (2012) 22:812–24. 10.1016/j.ccr.2012.11.003 23238016 PMC3984478

[13] Nagel D Spranger S Vincendeau M Grau M Raffegerst S Kloo B. Pharmacologic inhibition of MALT1 protease by phenothiazines as a therapeutic approach for the treatment of aggressive ABC-DLBCL. Cancer Cell. (2012) 22:825–37. 10.1016/j.ccr.2012.11.002 23238017

[14] Langel FD Jain NA Rossman JS Kingeter LM Kashyap AK Schaefer BC. Multiple protein domains mediate interaction between Bcl10 and MALT1. J Biol Chem. (2008) 283:32419–31. 10.1074/jbc.M800670200 18806265 PMC2583291

[15] Sommer K Guo B Pomerantz JL Bandaranayake AD Moreno-Garcia ME Ovechkina YL. Phosphorylation of the CARMA1 linker controls NF-kappaB activation. Immunity. (2005) 23:561–74. 10.1016/j.immuni.2005.09.014 16356855

[16] Matsumoto R Wang D Blonska M Li H Kobayashi M Pappu B. Phosphorylation of CARMA1 plays a critical role in T Cell receptor-mediated NF-kappaB activation. Immunity. (2005) 23:575–85. 10.1016/j.immuni.2005.10.007 16356856

[17] Li S Yang X Shao J Shen Y. Structural insights into the assembly of CARMA1 and BCL10. PLoS ONE. (2012) 7:e42775. 10.1371/journal.pone.0042775 22880103 PMC3411838

[18] Knies N Alankus B Weilemann A Tzankov A Brunner K Ruff T. Lymphomagenic CARD11/BCL10/MALT1 signaling drives malignant B-cell proliferation via cooperative NF-kappaB and JNK activation. Proc Natl Acad Sci USA. (2015) 112:E7230–8. 10.1073/pnas.1507459112 26668357 PMC4702996

[19] Sun L Deng L Ea CK Xia ZP Chen ZJ. The TRAF6 ubiquitin ligase and TAK1 kinase mediate IKK activation by BCL10 and MALT1 in T lymphocytes. Mol Cell. (2004) 14:289–301. 10.1016/S1097-2765(04)00236-9 15125833

[20] Oeckinghaus A Wegener E Welteke V Ferch U Arslan SC Ruland J. Malt1 ubiquitination triggers NF-kappaB signaling upon T-cell activation. EMBO J. (2007) 26:4634–45. 10.1038/sj.emboj.7601897 17948050 PMC2080808

[21] Wu CJ Ashwell JD. NEMO recognition of ubiquitinated Bcl10 is required for T cell receptor-mediated NF-kappaB activation. Proc Natl Acad Sci USA. (2008) 105:3023–8. 10.1073/pnas.0712313105 18287044 PMC2268578

[22] Ruland J Duncan GS Elia A del Barco Barrantes I Nguyen L Plyte S. Bcl10 is a positive regulator of antigen receptor-induced activation of NF-kappaB and neural tube closure. Cell. (2001) 104:33–42. 10.1016/S0092-8674(01)00189-1 11163238

[23] Egawa T Albrecht B Favier B Sunshine MJ Mirchandani K O'Brien W. Requirement for CARMA1 in antigen receptor-induced NF-kappa B activation and lymphocyte proliferation. Curr Biol. (2003) 13:1252–8. 10.1016/S0960-9822(03)00491-3 12867038

[24] Rebeaud F Hailfinger S Posevitz-Fejfar A Tapernoux M Moser R Rueda D. The proteolytic activity of the paracaspase MALT1 is key in T cell activation. Nat Immunol. (2008) 9:272–81. 10.1038/ni1568 18264101

[25] Coornaert B Baens M Heyninck K Bekaert T Haegman M Staal J. T cell antigen receptor stimulation induces MALT1 paracaspase-mediated cleavage of the NF-kappaB inhibitor A20. Nat Immunol. (2008) 9:263–71. 10.1038/ni1561 18223652

[26] Staal J Driege Y Bekaert T Demeyer A Muyllaert D Van Damme P. T-cell receptor-induced JNK activation requires proteolytic inactivation of CYLD by MALT1. EMBO J. (2011) 30:1742–52. 10.1038/emboj.2011.85 21448133 PMC3101995

[27] Hailfinger S Nogai H Pelzer C Jaworski M Cabalzar K Charton JE. Malt1-dependent RelB cleavage promotes canonical NF-kappaB activation in lymphocytes and lymphoma cell lines. Proc Natl Acad Sci USA. (2011) 108:14596–601. 10.1073/pnas.1105020108 21873235 PMC3167514

[28] Jeltsch KM Hu D Brenner S Zoller J Heinz GA Nagel D. Cleavage of roquin and regnase-1 by the paracaspase MALT1 releases their cooperatively repressed targets to promote T(H)17 differentiation. Nat Immunol. (2014) 15:1079–89. 10.1038/ni.3008 25282160

[29] Uehata T Iwasaki H Vandenbon A Matsushita K Hernandez-Cuellar E Kuniyoshi K. Malt1-induced cleavage of regnase-1 in CD4(+) helper T cells regulates immune activation. Cell. (2013) 153:1036–49. 10.1016/j.cell.2013.04.034 23706741

[30] Klein T Fung SY Renner F Blank MA Dufour A Kang S. The paracaspase MALT1 cleaves HOIL1 reducing linear ubiquitination by LUBAC to dampen lymphocyte NF-kappaB signalling. Nat Commun. (2015) 6:8777. 10.1038/ncomms9777 26525107 PMC4659944

[31] Elton L Carpentier I Staal J Driege Y Haegman M Beyaert R MALT1 cleaves the E3 ubiquitin ligase HOIL-1 in activated T cells, generating a dominant negative inhibitor of LUBAC-induced NF-kappaB signaling. FEBS J. (2016) 283:403–12. 10.1111/febs.13597 26573773

[32] Douanne T Gavard J Bidere N. The paracaspase MALT1 cleaves the LUBAC subunit HOIL1 during antigen receptor signaling. J Cell Sci. (2016) 129:1775–80. 10.1242/jcs.185025 27006117

[33] Baens M Bonsignore L Somers R Vanderheydt C Weeks SD Gunnarsson J. MALT1 auto-proteolysis is essential for NF-kappaB-dependent gene transcription in activated lymphocytes. PLoS ONE. (2014) 9:e103774. 10.1371/journal.pone.0103774 25105596 PMC4126661

[34] Yamasoba D Sato K Ichinose T Imamura T Koepke L Joas S. N4BP1 restricts HIV-1 and its inactivation by MALT1 promotes viral reactivation. Nat Microbiol. (2019) 4:1532–44. 10.1038/s41564-019-0460-3 31133753

[35] Mc Guire C Elton L Wieghofer P Staal J Voet S Demeyer A. Pharmacological inhibition of MALT1 protease activity protects mice in a mouse model of multiple sclerosis. J Neuroinflammation. (2014) 11:124. 10.1186/1742-2094-11-124 25043939 PMC4112826

[36] Rosenbaum M Gewies A Pechloff K Heuser C Engleitner T Gehring T. Bcl10-controlled Malt1 paracaspase activity is key for the immune suppressive function of regulatory T cells. Nat Commun. (2019) 10:2352. 10.1038/s41467-019-10203-2 31138793 PMC6538646

[37] Di Pilato M Kim EY Cadilha BL Prussmann JN Nasrallah MN Seruggia D. Targeting the CBM complex causes Treg cells to prime tumours for immune checkpoint therapy. Nature. (2019) 570:112–6. 10.1038/s41586-019-1215-2 31092922 PMC6656391

[38] McKinnon ML Rozmus J Fung SY Hirschfeld AF Del Bel KL Thomas L. Combined immunodeficiency associated with homozygous MALT1 mutations. J Allergy Clin Immunol. (2013) 133:1458–62, 1462.e1–7. 10.1016/j.jaci.2013.10.045 24332264

[39] Jabara HH Ohsumi T Chou J Massaad MJ Benson H Megarbane A. A homozygous mucosa-associated lymphoid tissue 1 (MALT1) mutation in a family with combined immunodeficiency. J Allergy Clin Immunol. (2013) 132:151–8. 10.1016/j.jaci.2013.04.047 23727036 PMC3700575

[40] Punwani D Wang H Chan AY Cowan MJ Mallott J Sunderam U. Combined immunodeficiency due to MALT1 mutations, treated by hematopoietic cell transplantation. J Clin Immunol. (2015) 35:135–46. 10.1007/s10875-014-0125-1 25627829 PMC4352191

[41] Charbit-Henrion F Jeverica AK Begue B Markelj G Parlato M Avcin SL. Deficiency in mucosa-associated lymphoid tissue lymphoma translocation 1: a novel cause of IPEX-like syndrome. J Pediatr Gastroenterol Nutr. (2017) 64:378–84. 10.1097/MPG.0000000000001262 27253662

[42] Wiegmann H Reunert J Metze D Marquardt T Engel T Kunde V Refining the dermatological spectrum in primary immunodeficiency: MALT1 deficiency mimicking Netherton- and Omenn syndrome. Br J Dermatol. (2019). 10.1111/bjd.18091. [Epub ahead of print]. 31049936

[43] Frizinsky S Rechavi E Barel O Najeeb RH Greenberger S Lee YN. Novel MALT1 mutation linked to immunodeficiency, immune dysregulation, and an abnormal T cell receptor repertoire. J Clin Immunol. (2019) 39:401–13. 10.1007/s10875-019-00629-0 31037583

[44] Jaworski M Marsland BJ Gehrig J Held W Favre S Luther SA. Malt1 protease inactivation efficiently dampens immune responses but causes spontaneous autoimmunity. EMBO J. (2014) 33:2765–81. 10.15252/embj.201488987 25319413 PMC4282555

[45] Yu JW Hoffman S Beal AM Dykon A Ringenberg MA Hughes AC. MALT1 protease activity is required for innate and adaptive immune responses. PLoS ONE. (2015) 10:e0127083. 10.1371/journal.pone.0127083 25965667 PMC4428694

[46] Bornancin F Renner F Touil R Sic H Kolb Y Touil-Allaoui I. Deficiency of MALT1 paracaspase activity results in unbalanced regulatory and effector T and B cell responses leading to multiorgan inflammation. J Immunol. (2015) 194:3723–34. 10.4049/jimmunol.1402254 25762782

[47] Gewies A Gorka O Bergmann H Pechloff K Petermann F Jeltsch KM. Uncoupling Malt1 threshold function from paracaspase activity results in destructive autoimmune inflammation. Cell Rep. (2014) 9:1292–305. 10.1016/j.celrep.2014.10.044 25456129

[48] Demeyer A Skordos I Driege Y Kreike M Hochepied T Baens M. MALT1 proteolytic activity suppresses autoimmunity in a T cell intrinsic manner. Front Immunol. (2019) 10:1898. 10.3389/fimmu.2019.01898 31474984 PMC6702287

[49] Cheng L Deng N Yang N Zhao X Lin X. Malt1 protease is critical in maintaining function of regulatory T cells and may be a therapeutic target for antitumor immunity. J Immunol. (2019) 202:3008–19. 10.4049/jimmunol.1801614 30979818

[50] Mc Guire C Wieghofer P Elton L Muylaert D Prinz M Beyaert R. Paracaspase MALT1 deficiency protects mice from autoimmune-mediated demyelination. J Immunol. (2013) 190:2896–903. 10.4049/jimmunol.1201351 23401595

[51] Brüstle A Brenner D Knobbe-Thomsen CB Cox M Lang PA Lang KS. MALT1 is an intrinsic regulator of regulatory T cells. Cell Death Differ. (2017) 24:1214–23. 10.1038/cdd.2015.104 26405015 PMC5584480

[52] Walker LS Sansom DM. The emerging role of CTLA4 as a cell-extrinsic regulator of T cell responses. Nat Rev Immunol. (2011) 11:852–63. 10.1038/nri3108 22116087

[53] Walker LS Sansom DM. Confusing signals: recent progress in CTLA-4 biology. Trends Immunol. (2015) 36:63–70. 10.1016/j.it.2014.12.001 25582039 PMC4323153

[54] Wang CJ Kenefeck R Wardzinski L Attridge K Manzotti C Schmidt EM. Cutting edge: cell-extrinsic immune regulation by CTLA-4 expressed on conventional T cells. J Immunol. (2012) 189:1118–22. 10.4049/jimmunol.1200972 22753931 PMC3442233

[55] Tai X Van Laethem F Pobezinsky L Guinter T Sharrow SO Adams A. Basis of CTLA-4 function in regulatory and conventional CD4(+) T cells. Blood. (2012) 119:5155–63. 10.1182/blood-2011-11-388918 22403258 PMC3369608

[56] Corse E Allison JP. Cutting edge: CTLA-4 on effector T cells inhibits in trans. J Immunol. (2012) 189:1123–7. 10.4049/jimmunol.1200695 22753941

[57] Geha RS Jabara HH Brodeur SR. The regulation of immunoglobulin E class-switch recombination. Nat Rev Immunol. (2003) 3:721–32. 10.1038/nri1181 12949496

[58] Lou H Lu J Choi EB Oh MH Jeong M Barmettler S. Expression of IL-22 in the skin causes Th2-biased immunity, epidermal barrier dysfunction, and pruritus via stimulating epithelial Th2 cytokines and the GRP pathway. J Immunol. (2017) 198:2543–55. 10.4049/jimmunol.1600126 28228560 PMC5360537

[59] Cianferoni A Spergel J. The importance of TSLP in allergic disease and its role as a potential therapeutic target. Expert Rev Clin Immunol. (2014) 10:1463–74. 10.1586/1744666X.2014.967684 25340427 PMC4332833

[60] Van Beek M Oravecz-Wilson KI Delekta PC Gu S Li X Jin X. Bcl10 links saturated fat overnutrition with hepatocellular NF-kB activation and insulin resistance. Cell Rep. (2012) 1:444–52. 10.1016/j.celrep.2012.04.006 22708078 PMC3375919

[61] Kingeter LM Schaefer BC. Loss of protein kinase C theta, Bcl10, or Malt1 selectively impairs proliferation and NF-kappa B activation in the CD4+ T cell subset. J Immunol. (2008) 181:6244–54. 10.4049/jimmunol.181.9.6244 18941215 PMC2630173

[62] Sehra S Yao Y Howell MD Nguyen ET Kansas GS Leung DY. IL-4 regulates skin homeostasis and the predisposition toward allergic skin inflammation. J Immunol. (2010) 184:3186–90. 10.4049/jimmunol.0901860 20147633 PMC2837507

[63] d'Hennezel E Bin Dhuban K Torgerson T Piccirillo CA. The immunogenetics of immune dysregulation, polyendocrinopathy, enteropathy, X linked (IPEX) syndrome. J Med Genet. (2012) 49:291–302. 10.1136/jmedgenet-2012-100759 22581967

[64] Ramsdell F Ziegler SF. FOXP3 and scurfy: how it all began. Nat Rev Immunol. (2014) 14:343–9. 10.1038/nri3650 24722479

[65] Wildin RS Smyk-Pearson S Filipovich AH. Clinical and molecular features of the immunodysregulation, polyendocrinopathy, enteropathy, X linked (IPEX) syndrome. J Med Genet. (2002) 39:537–45. 10.1136/jmg.39.8.537 12161590 PMC1735203

[66] Godfrey VL Wilkinson JE Russell LB. X-linked lymphoreticular disease in the scurfy (sf) mutant mouse. Am J Pathol. (1991) 138:1379–87. 2053595 PMC1886400

[67] Ju ST Sharma R Gaskin F Kung JT Fu SM. The biology of autoimmune response in the scurfy mice that lack the CD4+Foxp3+ regulatory T-cells. Biology. (2012) 1:18–42. 10.3390/biology1010018 24832045 PMC4011033

[68] Lahl K Mayer CT Bopp T Huehn J Loddenkemper C Eberl G. Nonfunctional regulatory T cells and defective control of Th2 cytokine production in natural scurfy mutant mice. J Immunol. (2009) 183:5662–72. 10.4049/jimmunol.0803762 19812199

[69] Fontenot JD Gavin MA Rudensky AY. Foxp3 programs the development and function of CD4+CD25+ regulatory T cells. Nat Immunol. (2003) 4:330–6. 10.1038/ni904 12612578

[70] Bacchetta R Barzaghi F Roncarolo MG. From IPEX syndrome to FOXP3 mutation: a lesson on immune dysregulation. Ann N Y Acad Sci. (2016) 1417:5–22. 10.1111/nyas.13011 26918796

[71] Brunkow ME Jeffery EW Hjerrild KA Paeper B Clark LB Yasayko SA. Disruption of a new forkhead/winged-helix protein, scurfin, results in the fatal lymphoproliferative disorder of the scurfy mouse. Nat Genet. (2001) 27:68–73. 10.1038/83784 11138001

[72] Tian L Altin JA Makaroff LE Franckaert D Cook MC Goodnow CC. Foxp3(+) regulatory T cells exert asymmetric control over murine helper responses by inducing Th2 cell apoptosis. Blood. (2011) 118:1845–53. 10.1182/blood-2011-04-346056 21715314 PMC3158716

[73] Roesner LM Werfel T Heratizadeh A. The adaptive immune system in atopic dermatitis and implications on therapy. Expert Rev Clin Immunol. (2016) 12:787–96. 10.1586/1744666X.2016.1165093 26967382

[74] Fyhrquist N Lehtimaki S Lahl K Savinko T Lappetelainen AM Sparwasser T. Foxp3+ cells control Th2 responses in a murine model of atopic dermatitis. J Invest Dermatol. (2012) 132:1672–80. 10.1038/jid.2012.40 22402436

[75] Oyoshi MK Larson RP Ziegler SF Geha RS. Mechanical injury polarizes skin dendritic cells to elicit a T(H)2 response by inducing cutaneous thymic stromal lymphopoietin expression. J Allergy Clin Immunol. (2010) 126:976–84, 84.e1–5. 10.1016/j.jaci.2010.08.041 21050944 PMC3085022

[76] Leyva-Castillo JM Hener P Jiang H Li M. TSLP produced by keratinocytes promotes allergen sensitization through skin and thereby triggers atopic march in mice. J Invest Dermatol. (2013) 133:154–63. 10.1038/jid.2012.239 22832486

[77] Hirota T Takahashi A Kubo M Tsunoda T Tomita K Sakashita M. Genome-wide association study identifies eight new susceptibility loci for atopic dermatitis in the Japanese population. Nat Genet. (2012) 44:1222–6. 10.1038/ng.2438 23042114

[78] Ma CA Stinson JR Zhang Y Abbott JK Weinreich MA Hauk PJ Germline hypomorphic CARD11 mutations in severe atopic disease. Nat Genet. (2017) 49:1192–201. 10.1038/ng.3898 28628108 PMC5664152

[79] Dadi H Jones TA Merico D Sharfe N Ovadia A Schejter Y. Combined immunodeficiency and atopy caused by a dominant negative mutation in caspase activation and recruitment domain family member 11 (CARD11). J Allergy Clin Immunol. (2017) 141:1818–30.e2. 10.1016/j.jaci.2017.06.047 28826773

[80] Dorjbal B Stinson JR Ma CA Weinreich MA Miraghazadeh B Hartberger JM Hypomorphic caspase activation and recruitment domain 11 (CARD11) mutations associated with diverse immunologic phenotypes with or without atopic disease. Jo Allergy Clin Immunol. (2019) 143:1482–95. 10.1016/j.jaci.2018.08.013 PMC6395549 30170123

[81] Barnes MJ Krebs P Harris N Eidenschenk C Gonzalez-Quintial R Arnold CN Commitment to the regulatory T cell lineage requires CARMA1 in the thymus but not in the periphery. PLoS Biol. (2009) 7:e51 10.1371/journal.pbio.1000051 19260764 PMC2650725

[82] Altin JA Tian L Liston A Bertram EM Goodnow CC Cook MC. Decreased T-cell receptor signaling through CARD11 differentially compromises forkhead box protein 3-positive regulatory versus T(H)2 effector cells to cause allergy. J Allergy Clin Immunol. (2011) 127:1277–85.e5. 10.1016/j.jaci.2010.12.1081 21320717 PMC3189857

[83] Policheni A Horikawa K Milla L Kofler J Bouillet P Belz GT. CARD11 is dispensable for homeostatic responses and suppressive activity of peripherally-induced FOXP3+ regulatory T cells. Immunol Cell Biol. (2019) 97:740–52. 10.1111/imcb.12268 31087793

[84] Staal J Driege Y Haegman M Borghi A Hulpiau P Lievens L. Ancient origin of the CARD-coiled coil/Bcl10/MALT1-like paracaspase signaling complex indicates unknown critical functions. Front Immunol. (2018) 9:1136. 10.3389/fimmu.2018.01136 29881386 PMC5978004

[85] Peled A Sarig O Sun G Samuelov L Ma CA Zhang Y. Loss-of-function mutations in caspase recruitment domain-containing protein 14 (CARD14) are associated with a severe variant of atopic dermatitis. J Allergy Clin Immunol. (2019) 143:173–81.e10. 10.1016/j.jaci.2018.09.002 30248356

[86] Afonina IS Van Nuffel E Baudelet G Driege Y Kreike M Staal J. The paracaspase MALT1 mediates CARD14-induced signaling in keratinocytes. EMBO Rep. (2016) 17:914–27. 10.15252/embr.201642109 27113748 PMC5278603

[87] Van Nuffel E Schmitt A Afonina IS Schulze-Osthoff K Beyaert R Hailfinger S. CARD14-mediated activation of paracaspase MALT1 in keratinocytes: implications for psoriasis. J Invest Dermatol. (2017) 137:569–75. 10.1016/j.jid.2016.09.031 27939769

[88] Rodriguez CI Buchholz F Galloway J Sequerra R Kasper J Ayala R. High-efficiency deleter mice show that FLPe is an alternative to Cre-loxP. Nat Genet. (2000) 25:139–40. 10.1038/75973 10835623

[89] Betz UA Vosshenrich CA Rajewsky K Muller W. Bypass of lethality with mosaic mice generated by Cre-loxP-mediated recombination. Curr Biol. (1996) 6:1307–16. 10.1016/S0960-9822(02)70717-3 8939573

[90] Wolfer A Bakker T Wilson A Nicolas M Ioannidis V Littman DR. Inactivation of Notch 1 in immature thymocytes does not perturb CD4 or CD8T cell development. Nat Immunol. (2001) 2:235–41. 10.1038/85294 11224523

[91] Ramirez A Page A Gandarillas A Zanet J Pibre S Vidal M. A keratin K5Cre transgenic line appropriate for tissue-specific or generalized Cre-mediated recombination. Genesis. (2004) 39:52–7. 10.1002/gene.20025 15124227

