# Supplementary material for: MALT1-Deficient Mice Develop Atopic-Like Dermatitis Upon Aging
Source: Front Immunol. 2019 Oct 1;10:2330. doi: 10.3389/fimmu.2019.02330 (PMC6779721; doi:10.3389/fimmu.2019.02330)
Supplement: Supplementary file 1 [file Image_1.pdf]

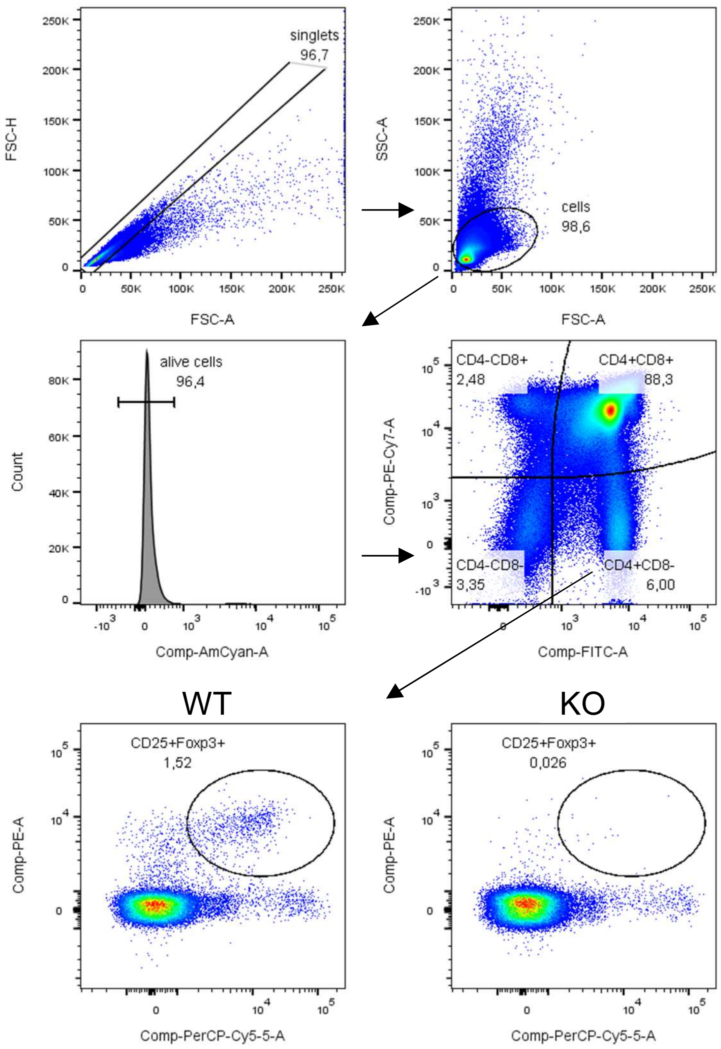

Supplementary Figure 1: Gating strategy for thymic Tregs ($CD4^+$ $CD8^-$ $CD25^+$ $FoxP3^+$) of *Malt1*-KO and WT mice

Singlets: FSC-A and FSC-H; cells: FSC-A and SSC-A; living cells: FSC-A and L/D eFluor506 negative; $CD4^+$ $CD8^-$ T cells: anti-CD4-FITC positive and anti-CD8-PE-Cy7 negative; Tregs ($Foxp3^+CD25^+CD4^+$ T cells): anti-Foxp3-PE and anti-CD25-PercPcy5.5 positive.

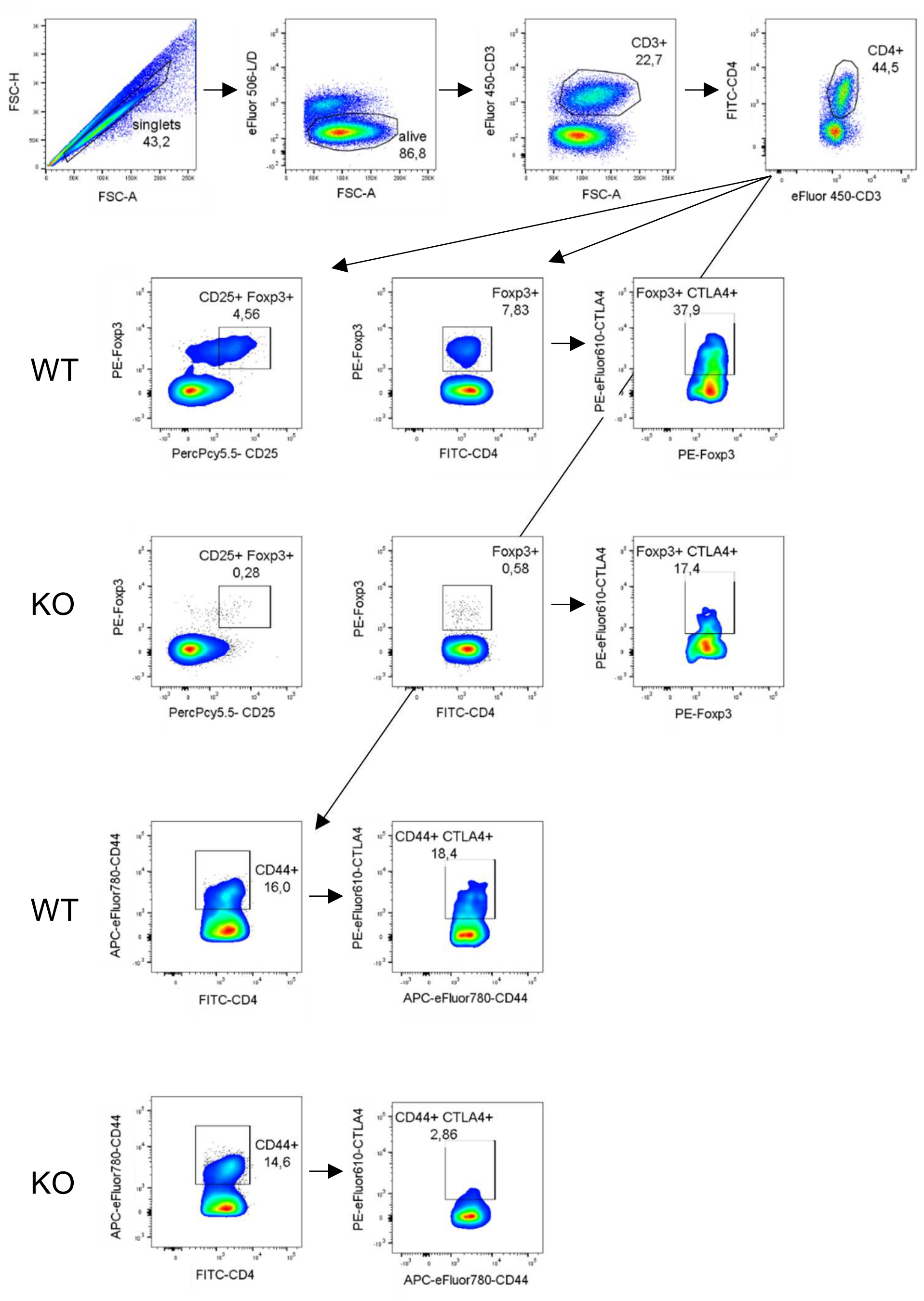

Supplementary Figure 2: Gating strategy for CTLA4$^+$ Foxp3$^+$ CD4$^+$ T cells and CTLA-4$^+$ CD44$^+$ CD4$^+$ T cells of *Malt1*-KO and WT mice

Singlets: FSC-A and FSC-H; cells: FSC-A and SSC-A; living cells: FSC-A and L/D eFluor506 negative; CD4$^+$ T cells: anti-CD3-eFluor and anti-CD4-FITC positive; Tregs (Foxp3$^+$ CD25$^+$ CD4$^+$ T cells): anti-Foxp3-PE and anti-CD25-PercPcy5.5 positive; surface CTLA4$^+$ Tregs: anti-Foxp3-PE and anti-CTLA4-PEeFluor610; surface CTLA4$^+$ CD44$^+$ CD4$^+$ T cells: anti-CD44-APC-eFluor780 and anti-CTLA4-PEeFluor610 positive.

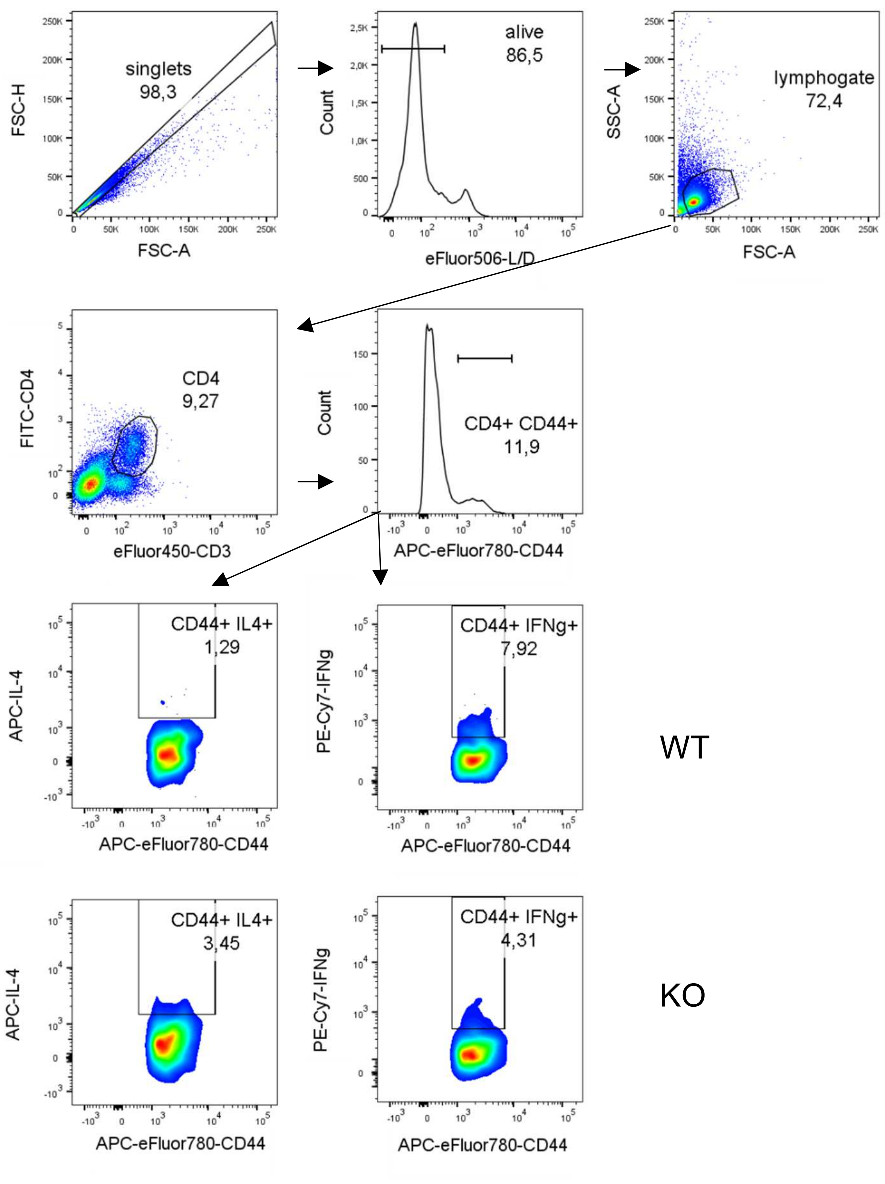

Supplementary Figure 3: Gating strategy for Th1 and Th2 cells of *Malt1*-KO and WT mice

Singlets: FSC-A and FSC-H; living cells: histogram L/D eFluor506 negative; cells (lymphogate): FSC-A and SSC-A; $CD4^+$ T cells: anti-CD3-eFluor and anti-CD4-FITC positive; $CD44^+$ $CD4^+$ T cells: histogram anti-CD44-APCeFluor780 positive; Th1 cells: anti-CD44-APCeFluor and anti-IFN-γ-PEcy7 positive; Th2 cells: anti-CD44-APCeFluor and anti-IL-4-APC positive.

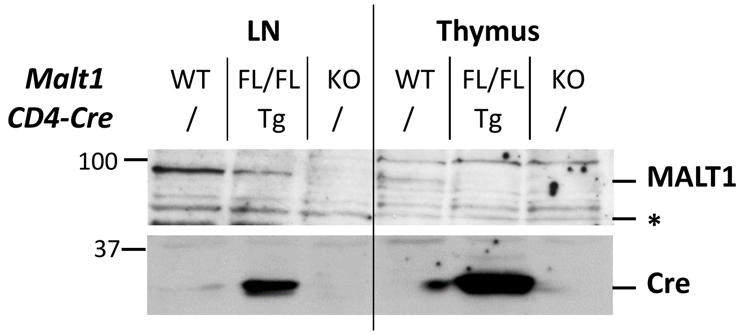

Supplementary Figure 4: Western blot showing *CD4-Cre* specific MALT1 loss in lymph nodes and thymus

Thymus (T cells) or lymph nodes (LN; mainly T cells and B cells) from *Malt1*-WT, *Malt1*$^{FL/FL}$ *CD4-Cre*$^{Tg/+}$ and *Malt1*-KO mice were lysed in E1A buffer and equal amounts of protein (100µg) were run on SDS-PAGE and blotted. The western blot was developed with an anti-MALT1 antibody recognizing murine MALT1 (top panel) and an anti-Cre recombinase antibody (lower panel). Aspecific band (*) was used as loading control.
